# Dengue Non-structural Protein 5 Polymerase Complexes With Promyelocytic Leukemia Protein (PML) Isoforms III and IV to Disrupt PML-Nuclear Bodies in Infected Cells

**DOI:** 10.3389/fcimb.2019.00284

**Published:** 2019-08-13

**Authors:** Federico Giovannoni, María Fatima Ladelfa, Martin Monte, David A. Jans, Peter Hemmerich, Cybele García

**Affiliations:** ^1^Laboratorio de Estrategias Antivirales, Departamento de Química Biológica, Facultad de Ciencias Exactas y Naturales, Universidad de Buenos Aires, Buenos Aires, Argentina; ^2^Instituto de Química Bioológica de la Facultad de Ciencias Exactas y Naturales (IQUIBICEN), CONICET-Universidad de Buenos Aires, Buenos Aires, Argentina; ^3^Laboratory of Molecular Oncology, Departamento de Química Biológica, Facultad de Ciencias Exactas y Naturales, Universidad de Buenos Aires, Buenos Aires, Argentina; ^4^Infection and Immunity Program, Department of Biochemistry and Molecular Biology, Monash Biomedicine Discovery Institute, Monash University, Clayton, VIC, Australia; ^5^Leibniz Institute on Aging, Jena, Germany

**Keywords:** dengue virus, non-structural protein 5 polymerase, promyelocytic leukemia protein, PML-NBs disruption, PML isoform

## Abstract

Dengue virus (DENV) threatens almost 70% of the world's population, with no therapeutic currently available. The severe, potentially lethal forms of DENV disease (dengue hemorrhagic fever/dengue shock syndrome) are associated with the production of high level of cytokines, elicited as part of the host antiviral response, although the molecular mechanisms have not been fully elucidated. We previously showed that infection by DENV serotype 2 (DENV2) disrupts promyelocytic leukemia (PML) gene product nuclear bodies (PML-NBs) after viral protein translation in infected cells. Apart from playing a key role as the nucleating agent in forming PML-NBs, PML has antiviral activity against various viruses, including DENV. The present study builds on this work, showing for the first time that all four DENV serotypes elicit PML-NB breakdown. Importantly, we show for the first time that of the nuclear localizing proteins of DENV, DENV non-structural protein (NS) 5 polymerase alone is sufficient to elicit PML-NB disassembly, in part through complexing with PML isoforms III and IV, but not other PML isoforms or other PML-NB components. The results raise the possibility that PML-NB disruption by nuclear localized NS5 contributes to DENV's suppression of the host antiviral response.

## Introduction

Dengue virus (DENV), a member of the genus Flavivirus within the family *Flaviviridae*, is the most significant cause of arthropod-borne viral disease in humans (Bhatt et al., 2013). DENV has a single, positive-stranded RNA genome of approximately 11 kb, with a single long open reading frame translated as a poly-protein, which is subsequently cleaved by a combination of cellular signal peptidases and the virally encoded protease NS2B/NS3 to yield three structural (C, prM and E) and seven non-structural (NS) proteins (NS1, NS2A, NS2B, NS3, NS4A, NS4B and NS5) (Ngono and Shresta, 2018).

Several positive-strand RNA viruses, including DENV, although replicating exclusively in the cytosol seemingly not requiring any nuclear intermediaries, interact with the host nucleus/host components of the nucleus (Weidman et al., 2003; Caly et al., 2015; Audsley et al., 2016; Jans and Martin, 2018) as part of the viral strategy to antagonize the host antiviral response. Viruses achieve this either by sequestering nuclear factors and/or trafficking their own proteins into the nucleus to impact host gene expression, and thereby attenuate the antiviral response (Hiscox, 2003; Caly et al., 2015; Audsley et al., 2016; Jans and Martin, 2018).

Three DENV viral proteins have been reported to access the host nucleus in infected cells. Of these, C (Tadano et al., 1989; Sangiambut et al., 2008) and NS5 (Pryor et al., 2007; Fraser et al., 2016) are known to localize in the infected cell nucleus, while NS3 was recently reported to be present in the host nucleus (Reyes-Ruiz et al., 2018). The polymerase NS5 is the largest (105 kDa) and most conserved protein encoded by the DENV genome, with critical roles in viral RNA synthesis through localization in membrane-associated replicase complexes in the cytoplasm of infected cells (Welsch et al., 2009). It has also been shown to shuttle between the nucleus and the cytoplasm antagonizing the antiviral response, by modulating IL-8 production and mRNA splicing in infected cells (Medin et al., 2005; Pryor et al., 2007; Ashour et al., 2009; Rawlinson et al., 2009; Morrison et al., 2013; De Maio et al., 2016); importantly, preventing NS5 nuclear access by small molecule inhibitors or mutation reduces infectious virus production significantly (Pryor et al., 2007; Wagstaff et al., 2012; Tay et al., 2013; Fraser et al., 2014).

Promyelocytic leukemia protein (PML) nuclear bodies (PML-NBs) are discrete punctate structures within the nucleus of mammalian cells. Although a small number of core proteins define the PML-NB (PML, DAXX, SP100, SUMO1-3), more than 100 other proteins have been reported to localize to PML-NBs in dynamic fashion (Van Damme et al., 2010), with certain PML-NB components known to undergo continuous exchange with the surrounding nucleoplasm (Weidtkamp-Peters et al., 2008). Significantly, the composition and morphology of PML-NBs can vary in response to cellular stress and virus infection (Dellaire and Bazett-Jones, 2004; Scherer and Stamminger, 2016), consistent with PML-NBs playing a role in key cellular functions, including the antiviral response (Hoischen et al., 2018).

Alternative splicing results in six major isoforms of the primary PML gene transcript, all of which localize in PML-NBs when overexpressed. Although all PML isoforms contain a conserved N-terminal region consisting of the RING, B-boxes, coiled-coil (RBCC) motifs, and the SUMO binding domain (SBD), differences in the C-terminal regions (Fagioli et al., 1992; Jensen et al., 2001; Nisole et al., 2013) are presumably the basis of isoform-specific roles. Various viruses are known to target specific isoforms (Nisole et al., 2013; Atwan et al., 2016), consistent with the idea that PML-NBs play key roles in the antiviral response (Scherer and Stamminger, 2016). The antiviral activity of PML-NBs has been shown for DNA viruses, for example, many of which alter/disorganize PML-NBs (Guccione et al., 2004; Geoffroy and Chelbi-Alix, 2011). Although less well studied, RNA viruses, some of which are of public health concern, have also been reported to use different strategies to counteract PML's antiviral function (Borden et al., 1998; Blondel et al., 2002; El McHichi et al., 2010).

We previously showed that PML appears to play an antiviral activity toward DENV-2 (Giovannoni et al., 2015). Here, we extend these studies to show for the first time that infection by all 4 DENV serotypes impacts on PML-NB structure, establishing that of the nuclear localizing DENV proteins, NS5 proteins but not NS3 or C are responsible, and that PML isoforms III and IV, but not other PML isoforms or PML-NB components, are the targets of NS5 action. Importantly, we show that PML-NB disruption by nuclear localized NS5 contributes to DENV suppression of the host antiviral response. This is the first report to highlight the key role of PML-NBs, through the NS5-PML III/IV interface, in DENV infection.

## Results

### PML Is an Antiviral Host Factor for All DENV Serotypes

**Figure 1:**
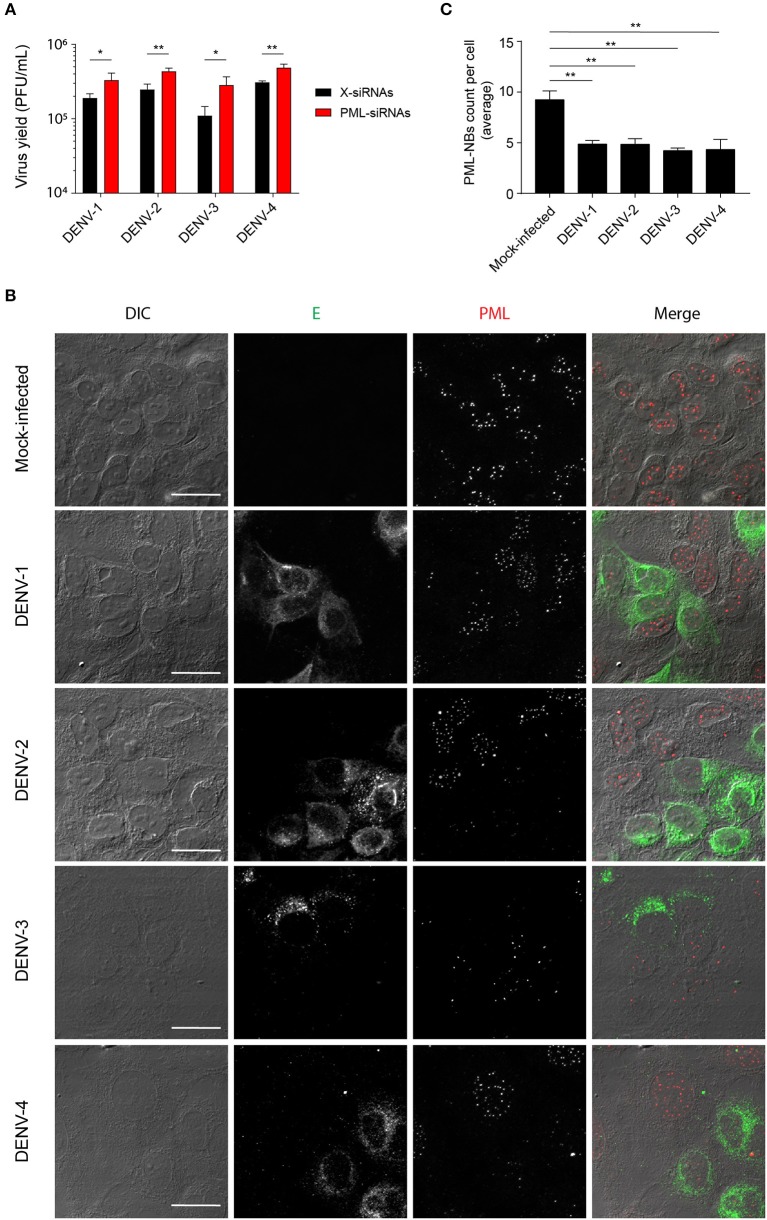
Infection with all 4 DENV serotypes can disrupt host cell PML-NBs. **(A)** A549 cells were transfected with PML-siRNAs or X-siRNAs (scrambled sequence) and then infected with one of DENV1-4 (MOI of 1) as indicated. Culture supernatants were harvested at 48 h post-infection (p.i.) and infectious virus particles quantified by plaque assay. Data represent the mean ± SD from three independent experiments. ^*^p < 0.05; ^**^p < 0.01 (Student's t test). **(B)** A549 cells were mock-infected or infected with one of the four DENV serotypes as indicated. Cells were fixed and stained at 48 h p.i. against DENV envelope glycoprotein E (green) and PML (red). Confocal images were acquired using an Olympus FV1000 setup. Differential interference contrast (DIC) images are shown on the left. Scale Bar: 20 μm. **(C)** Quantification of the average number of PML-NBs per nucleus was performed using FIJI. At least 50 cells per condition and per experiment were quantified. Data represent the mean ± SD from three independent experiments. ^**^p < 0.01 (one-way ANOVA followed by Tukey's *post-hoc* test).

We previously reported that PML plays an antiviral role in infection by DENV-2 (Giovannoni et al., 2015). Here we set out to extend this work, initially using siRNA to test the importance of PML for infection by other serotypes of DENV. To this end, cultures of A549 cells were treated with siRNA specific to PML or control scrambled siRNA and then infected with DENV-1-4, prior to determination of virus yields 48 h later using plaque assays. PML knock-down resulted in significantly (*p* < 0.05) higher infectious virus production for all DENV serotypes compared to the scrambled siRNA controls (Figure 1A). The infected cell cultures were also analyzed by confocal microscopy, with maximum intensity projections generated from Z-stacks (Figure 1B) enabling the number of PML-NBs per nucleus to be quantified (Figure 1C). In the case of all DENV serotypes, infection was found to significantly (*p* < 0.01) reduce the number of PML-NBs by ~2-fold (Figure 1C).

### DENV NS5 Is Sufficient to Promote PML-NB Disruption

**Figure 2:**
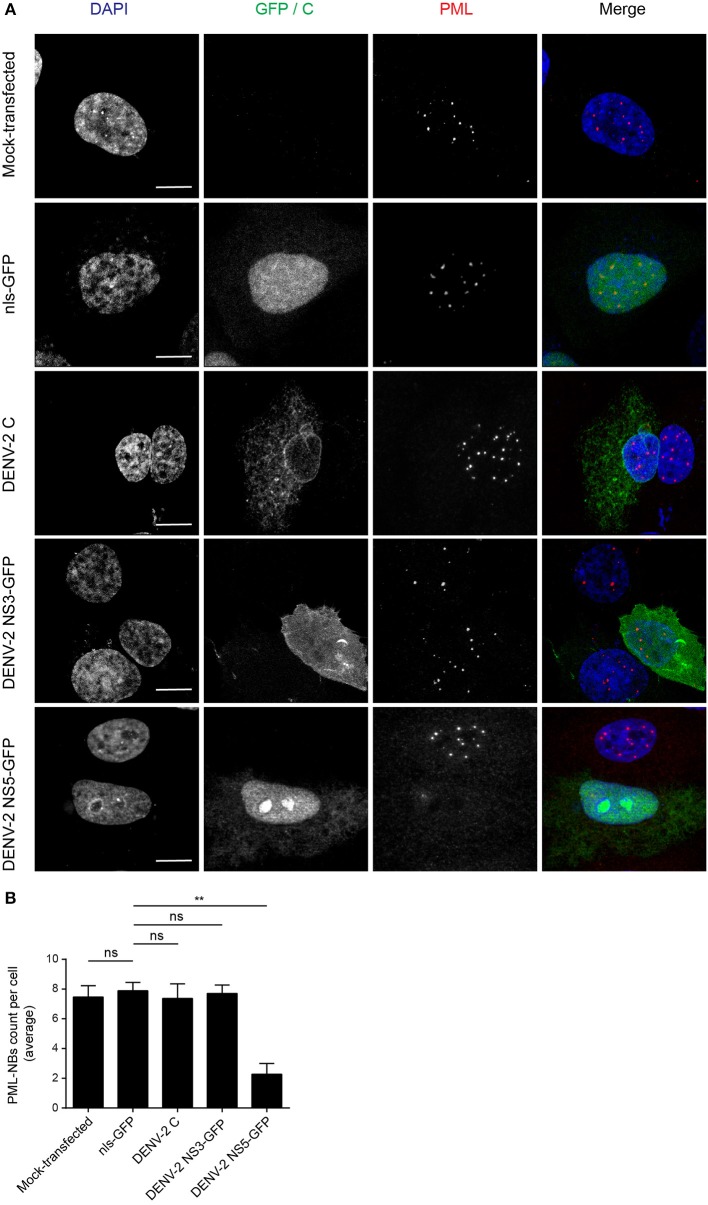
Ectopically expressed DENV-2 NS5 is sufficient to mediate PML-NBs disassembly. **(A)** A549 cells were mock-transfected or transfected with plasmids encoding nls-GFP, DENV-2C, DENV-2 NS3-GFP, or DENV-2 NS5-GFP (all green) as indicated. 24 h later, cells were fixed and stained for PML (red), and where appropriate, for C (green). Confocal images were acquired using a Zeiss LSM710 setup. Scale Bar: 10 μm. **(B)** Quantification of the average number of PML-NBs per nucleus was performed using FIJI. At least 50 cells per condition and per experiment were quantified. Data represent the mean ± SD from three independent experiments. Ns, not statistically significant; ^**^p < 0.01 (one-way ANOVA followed by Tukey's *post-hoc* test).

The DENV genome encodes three proteins (C, NS3, and NS5) that are able to localize in the nucleus of infected cells. In order to determine whether any of these may be directly responsible for the effects on PML-NBs, A549 cells were transfected to express green fluorescent protein (GFP)-tagged (NS3, NS5) or untagged (C protein) versions of the proteins derived from DENV-2. A vector expressing GFP fused to a nuclear localization signal (nls-GFP) that can localize in the nucleus served as a control. Confocal imaging (Figure 2A) revealed that the pattern of PML-NBs was not affected in cells expressing C, GFP-NS3, or nls-GFP, in stark contrast to cells expressing GFP-NS5, which showed significantly (*p* < 0.01) reduced numbers (70% lower—Figures 2A,B).

**Figure 3:**
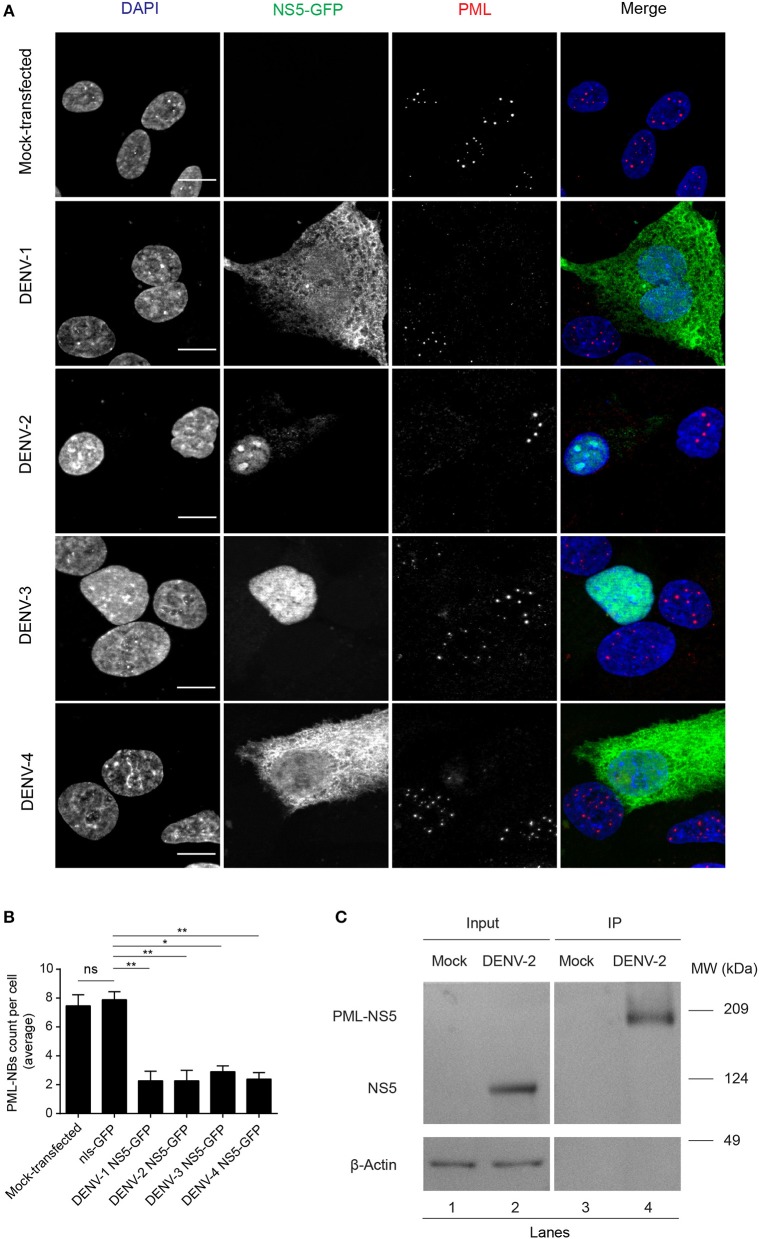
NS5 from DENV1-4 is sufficient to mediate PML-NBs disassembly. **(A)** A549 cells were transfected with plasmids encoding for NS5-GFP of DENV1-4 (green). 24 h later, cells were fixed and stained for PML (red). Images of optical sections were acquired using a Zeiss Apotome.2 setup. Scale Bar: 10 μm. **(B)** Quantification of the average number of PML-NBs per nucleus was as per the legend to Figure 2B. ns, not statistically significant; ^*^p < 0.05; ^**^p < 0.01 (one-way ANOVA followed by Tukey's *post-hoc* test). **(C)** A549 cells were mock-infected or infected with DENV-2 and 48 h p.i. they were harvested for immunoprecipitation against PML followed by Western blot against DENV-2 NS5. Lanes 1 and 2 correspond to whole cell lysates (Input) and 3 and 4 correspond to precipitates (IP). Images are representative of three independent experiments.

The number of PML-NBs was also analyzed in cell cultures transfected to express NS5-GFP proteins from the other DENV serotypes (Figure 3). GFP-tagged NS5 from DENV-1 and−4 was found in both cytoplasm and nucleus, whereas NS5 from DENV-2 and−3 was mainly nuclear, indicative of quantitative differences between DENV1-4 in the extent of NS5 nuclear localization (Figure 3A), in agreement with previous studies (Tay et al., 2013). Importantly, expression of NS5 from any of the four DENV serotypes significantly (*p* < 0.05) reduced the number of PML-NBs by about 70% compared to untransfected cells or those expressing nls-GFP (Figure 3B). Thus, NS5 from all 4 DENV serotypes is sufficient to disrupt PML-NBs even out of the context of the infected cell, indicating conservation of this property across all of the DENV serotypes.

To assess association of NS5 with PML, we used PML specific antibodies to perform co-immunoprecipitation from cell lysates prepared from A549 cells infected with DENV-2 (Figure 3C). Precipitates were extensively washed, and then eluates electrophoresed and subjected to Western blot analysis using anti-NS5 specific antibodies. Figure 3C shows that anti-PML antibodies were able to pull-down NS5 from the infected cell lysates but not from mock-infected cells (Figure 3C, compare lanes 4 and 3, respectively), with an electrophoretic band for the complex of PML and NS5 at the predicted molecular weight of ca. 170 kDa., indicating the viral protein is interacting with an specific PML isoform. Given the molecular weight of NS5 (95 kDa), complexation of PMLIII, IV, or V and NS5 may represent a key step for subsequent disassembly of PML-NBs.

### PML Isoforms III and IV Specifically Recruit NS5 Into NBs

**Figure 4:**
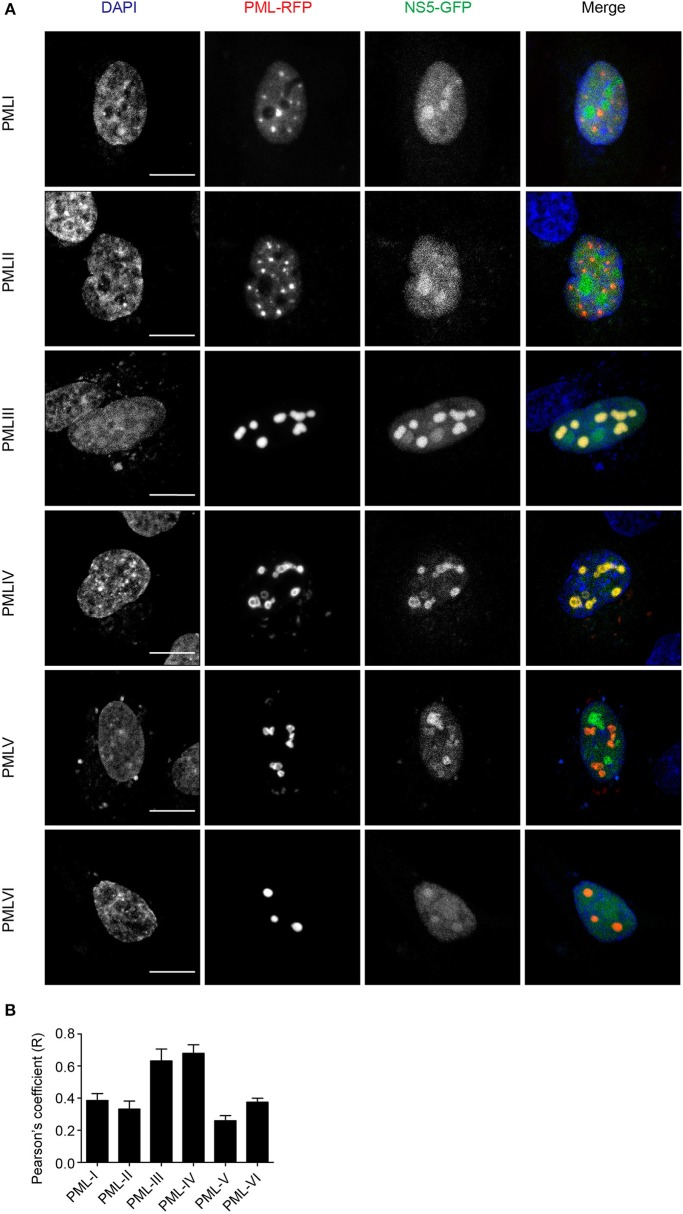
PMLIII-RFP and PMLIV-RFP colocalize with DENV-2 NS5-GFP. **(A)** A549 cells were co-transfected with vectors encoding one of the six PML-RFP isoforms and DENV-2 NS5-GFP as indicated. At 24 h p.t., cells were fixed and confocal images were acquired using a Zeiss LSM710 setup. Scale bar: 10 μm. **(B)** Quantification of the Pearson's coefficient was performed using FIJI. At least 10 cells per condition and per experiment were quantified. Data represent the mean ± SD from two independent experiments, performed in triplicate.

To assess which of the six nuclear PML isoforms (PML-I-VI) may be instrumental to attract NS5 into PML-NBs, A549 cells were co-transfected to express DENV-2 NS5-GFP together with one of PML isoforms I-VI fused to monomeric Red Fluorescent Protein (mRFP-PML), and observed by confocal microscopy. As shown in Figure 4, minimal co-localization was observed between NS5-GFP and mRFP-PML-I, -PML-II, -PML-V, or -PML-VI. In contrast, mRFP-PML-III and -PML-IV showed strong co-localization in PML-NBs (Figure 4A); this was supported by quantitative analysis for Pearson's co-localization coefficient, which revealed values approaching 0.7, compared to the other isoforms where the value was below 0.4 (Figure 4B).

**Figure 5:**
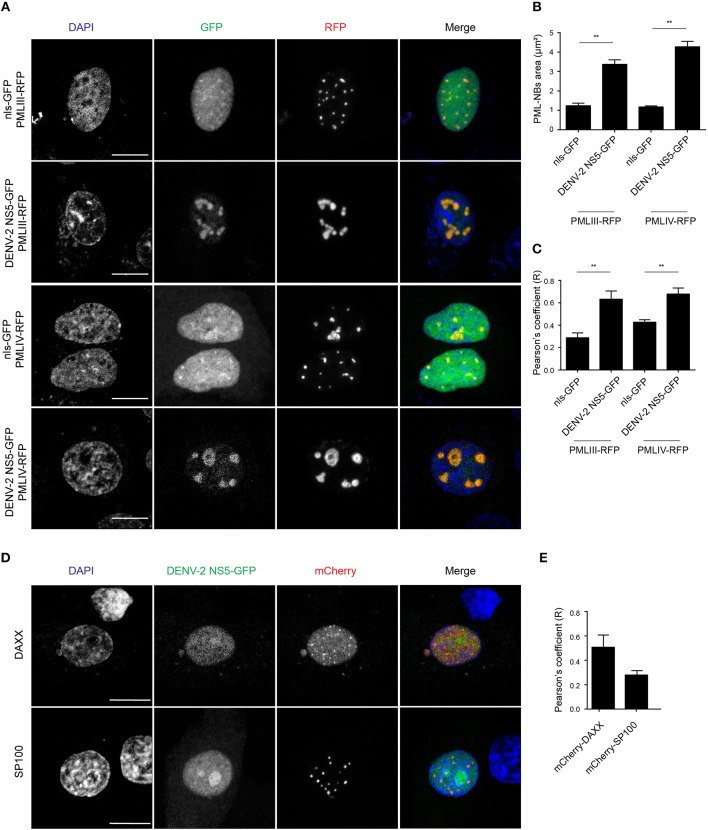
Accumulation of NS5-GFP in PML-NBs overexpressing PMLIII/-IV-RFP. **(A)** A549 cells were cotransfected with vectors encoding for DENV-2 NS5-GFP or nls-GFP, and PMLIII-RFP, PMLIV-RFP, or **(D)** mCherry-SP100 or mCherry-DAXX as indicated. At 24 h p.t., cells were fixed and confocal images were acquired using a Zeiss LSM710 setup. Scale bar: 10 μm. **(B)** Quantification of the mean PML-NB area in A549 cells (*n* > 20 for each condition and experiment) co-expressing DENV-2 NS5-GFP or nls-GFP and PMLIII- or IV-RFP was performed using FIJI. Data represent the mean ± SD from three independent experiments, each performed in triplicate. ^**^p < 0.01 (Student's *t* test) **(C,E)**. Quantification of the Pearson's coefficient was performed using FIJI. At least 20 cells per condition and per experiment were quantified. Data represent the mean ± SD from three independent experiments, each performed in triplicate. ^**^p < 0.01 (Student's *t* test).

In order to gain a more quantitative estimate of the co-localization, we compared NS5 recruitment by PML-III and -IV with that of overexpressed nls-GFP alone. As above, ectopic expression of either PML-III or -IV induced the accumulation of NS5-GFP in large PML-NB structures, in contrast to the discrete punctate pattern observed in the presence of co-expressed nls-GFP, which was diffusely nucleoplasmic (Figure 5A). Quantitative analysis showed that the average area of PML-NBs containing GFP-NS5 was significantly (*p* < 0.01) higher (3–4-fold) in cells co-expressing PML-III or -IV than in those co-expressing nls-GFP (Figure 5B). Importantly, significantly (*p* < 0.01) increased co-localization of (ca. 2-fold) NS5 and PML-III or –IV compared to nls-GFP was evident, as indicated by the estimation of Pearson's coefficient (Figure 5C). As negative controls, cells were also co-transfected to express NS5-GFP along with vectors encoding mCherry-tagged DAXX or SP100 (constitutive PML-NB components); there was no accumulation of NS5-GFP in PML-NBs in mCherry-SP100 or -DAXX overexpressing cells (Figures 5D,E). This is consistent with the idea that NB recruitment of NS5-GFP by the PML-III and IV isoforms is specific, and not a property of PML-NB components generally.

### Effect of NS5 Expression on PML Protein Levels

**Figure 6:**
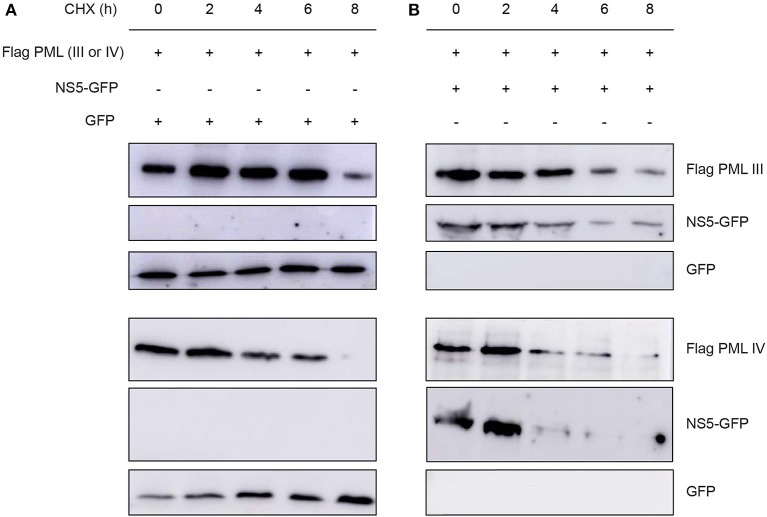
Effect of NS5 expression on PML protein levels. Western blot showing Flag-PMLIII or -PMLIV protein levels in HEK293 cells transfected to express GFP alone **(A)** or NS5-GFP **(B)** as indicated. The day after transfection, cells were incubated with cycloheximide (CHX) for 0–8 h, prior to cell lysis, gel electrophoresis, and Western blot analysis using the indicated antibodies. Images are representative of three independent experiments.

Next, we assessed the effect of NS5 expression on ectopically expressed PML protein levels in cells treated with the translation inhibitor cycloheximide. Cycloheximide induced a decrease in PML-III and –IV levels after about 6–8 h of treatment; NS5 co-expression appeared to accelerate this effect, with decreased PML-III and -IV levels after 4 h of treatment (Figure 6). This reduction in PML-III and -IV levels appeared to be independent of protein translation, suggesting that NS5 actively increases PML turnover.

### PML III and IV Inhibit DENV Replication

To put our results in physiological context, we tested the effect of ectopic expression of specific PML isoforms on DENV-2 infection. HEK-293 cells, extensively used in flavivirus research because of their ease of transfection and permissiveness to DENV infection (Chan and Gack, 2016; Aguirre et al., 2017), were transfected to express one of PML isoforms I-VI fused to RFP. 48 h later, cells were infected with DENV-2 (MOI = 1) and fixed 48 h post-infection, prior to immunofluorescence against the viral antigen E to assess viral replication. Figure 6A shows that HEK-293 cells ectopically expressing either RFP-tagged PML-III or -IV displayed a marked decrease in the expression of DENV-2 viral antigen compared to non-transfected or nls-GFP expressing cells. Overexpression of other PML isoforms or PML associated components DAXX or SP100 did not alter viral antigen expression. Cell culture supernatants were harvested to determine infectious virus production. Figure 6B shows that cells transfected with nls-GFP had a viral particle production similar to that of non-transfected cells. Ectopic expression of PML-I, -II, -V, -VI, or DAXX or SP100 did not significantly impact infectious virus production. In contrast, ectopic expression of either PML-III or -IV significantly (*p* < 0.01) decreased viral yield by 60% compared to the control (Figure 6B). These results strongly imply that PML isoforms III and IV, but not the other PML isoforms or PML components, appear to have specific antiviral activity toward DENV.

## Discussion

**Figure 7:**
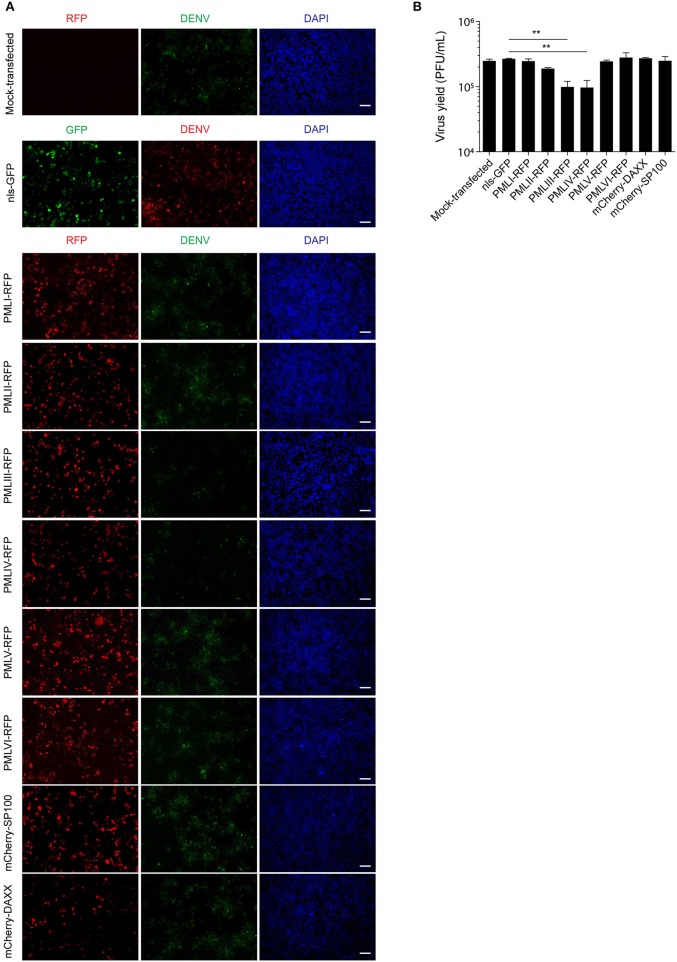
PMLIII and PMLIV reduce DENV-2 viral replication. **(A)** HEK-293 cells were mock-transfected or transfected with nls-GFP, PML-RFP (isoforms I to VI), mCherry-DAXX or mCherry-SP100. 48 h p.t. cells were infected with DENV-2 (MOI = 1) and 48 h later, fixed and stained for DENV envelope glycoprotein E (green or red, as indicated in the figure). Standard fluorescence microscopy images were acquired using an Olympus BX51 setup. Scale bar: 100 μm. **(B)** The antiviral effect of PMLIII or PMLIV on DENV-2 virus production was quantified by plaque assay. Data represent the mean ± SD from three independent experiments, performed in duplicate. ^**^p < 0.01 (one-way ANOVA followed by Tukey's *post-hoc* test).

Collectively, our results show for the first time that PML exerts antiviral activity against all 4 DENV serotypes, underlying PML's importance as an antiviral host factor in the case of infection by DENV. In particular, significant increments of viral yield in PML knock down infected cell cultures were detected (Figure 1A). In line with our previous results, microscopic observations revealed that PML-NBs are disrupted after DENV infection (Figures 1B,C), implying that DENV-induced PML-NB disruption may be a strategy to circumvent this cellular restriction point. Indeed, we show that expression of NS5 alone is sufficient to disrupt PML-NBs (Figures 2, 3A,B). Significantly, a complex between endogenous PML and NS5 could be detected in infected cell lysates (Figure 3C), which may represent a key step for PML-NB disassembly. Strikingly, PML-III and –IV showed localization in much larger nuclear bodies in the presence than in the absence of NS5, in contrast to other PML isoforms. These observations suggest that PML isoforms III and IV may uniquely recruit NS5 into PML-NBs, and/or alter PML-NB structure/composition (Figures 4, 5). In fact, degradation of PML after NS5 transfection was observed by different approaches confirming NS5 as the counteracting viral protein for PML antiviral activity (Figures 3, 6). Finally, we found that PML-III and –IV, but not other PML isoforms or other components of PML-NB, act to reduce infectious virus production (Figure 7). PML-IV has been implicated in the restriction of viruses such as the encephalomyocarditis virus, where it sequesters the viral polymerase in PML-NBs, thereby blocking its activity (Maroui et al., 2011), and varicella-zoster virus (Reichelt et al., 2011), where PML IV uniquely sequesters the viral capsid protein into PML-NBs. Whether PML exerts its antiviral effect against DENV through sequestering NS5 in PML-NBs and preventing replication remains to be determined. Clearly, however, NS5's role in targeting PMLIII/IV and thereby PML-NBs seems highly significant for the evasion of intrinsic and innate immunity.

The concept of intrinsic immunity was first introduced in 2004 (Bieniasz, 2004). It was proposed to be mediated by host restriction factors, constitutively expressed proteins with antiviral activity (Kluge et al., 2015). Restriction factors have been proposed to fulfill four main criteria (Duggal and Emerman, 2012); it must restrict virus production, the virus must have a strategy to circumvent it, it must be IFN induced, and its gene must show evidence of positive selection. Although the PML gene does not show evidence of positive selection (Malfavon-Borja et al., 2013), we have previously shown that it both exerts an antiviral effect against DENV and is induced by IFN (Giovannoni et al., 2015). The results here formally confirm that PML's antiviral activity is counteracted by a viral protein (NS5). Clearly, irrespective of whether PML can be formally delineated as a host restriction factor for viral infection in this context, PML is clearly an interesting candidate for the development of future therapies against DENV and potentially other flaviviruses.

## Materials and Methods

### Cells and Viruses

A549 (ATCC, CCL-185) and Vero (ATCC, CCL-81) cells were grown in Eagle's minimum essential medium (MEM, GIBCO) supplemented with 10 and 5% fetal bovine serum (GIBCO), respectively, and 50 μg/mL gentamycin (Thermo Fisher Scientific).

HEK-293 cells (ATCC, CRL-1573) were grown in DMEM/F12 (GIBCO) supplemented with 10% fetal bovine serum and 50 μg/mL gentamycin.

C6/36 mosquito cell line from *Aedes albopictus*, was cultured in L-15 medium (Leibovitz, GIBCO) supplemented with 0.3 % tryptose phosphate broth (Sigma), 0.02% glutamine (Sigma), 1% MEM non-essential amino acids solution (GIBCO) and 10% fetal bovine serum.

DENV strains DENV-1 (Hawaii), DENV-2 (New Guinea C), DENV-3 (H87), and DENV-4 (8,124) were provided by Dr. A.S. Mistchenko (Hospital de Ninos Dr. Ricardo Gutierrez, Buenos Aires, Argentina). Virus stocks were prepared by infecting C6/36 cells with the appropriate DENV serotype at a MOI of 0.1. Supernatants from day 3–7 p.i. were harvested, clarified, filtered, and stored at −80°C until use. Quantification of the virus titers was performed by a plaque assay (see Section 4.4 below). The titer was expressed as the number of plaque-forming units (PFU) per milliliter.

### siRNA, Plasmid Constructs and Transfections

For siRNA transfection, A549 cells were seeded into 24-well microplates at a density of 1^*^10^5^ cells per well prior to transfection using Lipofectamine 2000 (Thermo Fisher Scientific) the following day with 50 nM siRNA (sc-36283, Santa Cruz Biotechnology) following the manufacturer's specifications. Briefly, 25 pmol siRNA and 1 μl Lipofectamine 2000 was prepared in 100 μl of Opti-MEM (Thermo Fisher Scientific), incubated at room temperature for 15 min, and then added to the cells. 48 h post transfection, cells were used for subsequent analysis (viral infection/plaque assays). Non-targeting scrambled siRNA (X-siRNA, sc-37007, Santa Cruz Biotechnology) was used as a negative control. siRNA knockdown efficiency (ca. 60%) was confirmed by qRT-PCR and indirect immunofluorescence as previously (Giovannoni et al., 2015).

All plasmid constructs used have been described previously, including nls-GFP, pmRFP–PML isoforms I to VI, mCherry-SP100, mCherry-DAXX (Weidtkamp-Peters et al., 2008; Brand et al., 2010; Ulbricht et al., 2012), DENV1-4 NS5-GFP (Tay et al., 2013), DENV-2 C (Samsa et al., 2009), and pCMV5-Flag PMLIII, and pCMV5-Flag PMLIV (Peche et al., 2012).

For plasmid transfection, A549 cells were seeded into 24-well microplates at a density of 1^*^10^5^ cells per well and transfected the following day. Briefly, transfection mixture containing 0.5 μg of plasmid DNA and 1.5 μl Lipofectamine 2000 was prepared in 100 μl of Opti-MEM and incubated at room temperature for 15 min before addition to the cells. 24 h post transfection, cells were used for downstream applications (e.g., Indirect immunofluorescence and microscopy). Co-transfections were performed by using 0.25 μg of each plasmid DNA.

Plasmid DNA transfection into HEK-293 cells for evaluating the antiviral effect of PML isoforms and PML-NBs components was performed using Fugene HD (Roche). Briefly, HEK-293 cells were seeded into 24-well plates at a density of 8^*^10^4^ cells per well and transfected the following day. Transfection mixture containing 0.5 μg of plasmid DNA and 1.75 μl of Fugene HD was prepared in 100 μl of Opti-MEM and incubated at room temperature for 15 min before addition to the cells. 48 h later, cells were used for subsequent assays (viral infection/plaque assays).

In all cases, mock transfections for either siRNA or plasmid DNA were performed by adding the mix of transfection reagent and Opti-MEM lacking siRNA/DNA to the cells.

### Viral Infection

A549 and HEK-293 were infected at a MOI of 1. The cells were incubated for 1 h at 37°C prior to removal of the viral inoculum and washing three times with PBS. Finally, fresh medium supplemented with 2% fetal bovine serum was added. Mock infections were performed by incubating the cells with fresh medium instead of viral inoculum for 1 h as described.

### Plaque Assay

Vero cells (2^*^10^5^ cells per well) were seeded into 24-well microplates and grown overnight. 10-fold dilutions of samples containing virus were added to monolayers of confluent Vero cells at 37°C for 1 h. Following incubation, the inoculum was removed, and monolayers overlaid with 1 ml of MEM containing 1% methylcellulose. The cells were incubated at 37°C for 7 days and fixed using 4% formaldehyde. Finally, plaques were stained with 0.1% crystal violet in 20% ethanol and counted.

### Indirect Immunofluorescence and Microscopy

Cell monolayers (seeded at 2^*^10^5^ cells per well) grown on coverslips were washed with cold PBS and fixed with 4% formaldehyde for 10 min at room temperature and permeabilized using 0.25% Triton X-100 for 3 min. Primary antibodies and dilutions (in PBS) used were as follows: anti-PML mAb (1:500, sc-966, Santa Cruz Biotechnology), rabbit anti-PML (ABD-030, Jena Biosciences, and sc-5621, Santa Cruz Biotechnology), anti-E of DENV-1, DENV-2, and DENV-3 mAb (1:300, ab9202, Abcam), anti-E of DENV-4 mAb (1:100, NR-15534, BEI Resources, kind gift of Dr. Itati Ibañez), rabbit anti-C DENV-2 (1:500, kind gift of Dr. Andrea Gamarnik). After washing steps with PBS, secondary antibodies used were: Alexa Fluor 488-anti–mouse IgG (1:400, Thermo Fisher Scientific), Cy3-, or Cy5-anti–rabbit IgG (1:600, Dianova). Finally, coverslips were mounted in Prolong Gold mounting medium with 4',6-diamidino-2-phenylindole (DAPI) (Thermo Fisher Scientific). Alternatively, cells were stained with DAPI and mounted in a glycerol solution containing 1,4-diazabicyclo[2, 2, 2]octane (DABCO). For microscopy of fixed cells different setups were used, including a laser-scanning confocal microscope LSM 710/ConfoCor 3 (Carl Zeiss) or Olympus FV1000 (Olympus), a structured illumination microscope Zeiss Apotome.2 (Carl Zeiss) or a standard widefield fluorescence microscope Olympus BX51 (Olympus). The setup used for acquiring images is described in each figure legend.

### Image Analysis

Quantification of the average number of PML-NBs per cell nucleus was performed using the Fiji distribution of ImageJ (Schindelin et al., 2012). Briefly, images (Z-stacks) of random fields of view were acquired and maximum intensity projections generated. Each cell to be counted was selected and the *Find Maxima* tool was used; at least 50 cells per condition were analyzed in this way to give an average estimation. For co-localization analysis, Pearson's correlation coefficients were calculated using the Coloc2 Plugin on Fiji, with at least 20 cells per condition were analyzed in this way to give an average estimation (Manders et al., 1993). For determination of PML-NB area, maximum intensity projections were converted to grayscale, thresholded and the *Analyze Particles* tool was used; with at least 20 cells per condition analyzed in this way to give an average estimation.

### Immunoprecipitation and Western Blot

A549 cells (~5 × 10^5^) were infected with DENV-2 at an MOI of 1. 48 h after infection, cells were harvested with 1% CHAPS in TBS supplemented with protease inhibitor cocktail (Sigma). Samples were frozen at −80°C and thawed for three times. After this, the samples were centrifuged (10 min, 350 g) and supernatants were incubated for 1 h at 37°C with 2 μg of anti-PML mAb (sc-966, Santa Cruz Biotechnology). Antigen-antibody complexes were immobilized by rotation for 2 h at 4°C with Protein A-Agarose Beads (Roche). The complexes were pelleted (10 min, 350 g) and the supernatant was removed. The complexes were then washed three times with the same buffer used for the immunoprecipitation. After the last wash, complexes were resuspended in 50 μl of sample buffer without β-mercaptoethanol and boiled for 5 min. Finally, samples were centrifuged and supernatants were subjected to SDS-PAGE and Western blot.

For Western blot analysis, whole cell lysates (input) and precipitates (IP) and prestained molecular markers (Bio-Rad 161-0318) were separated by a 12% polyacrylamide gel and transferred to a PVDF membrane (PerkinElmer) using a semi-dry system (Bio-rad). Membranes were blocked with TBS containing 0.1% Tween (TBS-T) and 5% skimmed milk at 37°C for 1 h. Then, membranes were washed with TBS-T and incubated with rabbit anti-NS5 DENV-2 (1:1,000, kind gift of Dr. Andrea Gamarnik) in blocking solution at 4°C overnight. After rinsing in TBS-T, anti-rabbit IgG HRP-linked antibody (1:2,500, W4011, Promega) was diluted in blocking solution and incubated with the membranes for 1 h at 37°C. Protein bands were visualized by a chemiluminescence detection system using Western Lightning ECL (PerkinElmer). Finally, this protocol was repeated for β-actin using anti- β-actin mAb (1:1,000, #4,967, Cell Signaling) and anti-mouse IgG HRP-linked antibody (1:4,000, A9044, Sigma).

### Cycloheximide Chase Analysis of Protein Degradation

For cycloheximide chase analysis, HEK-293T cells (4^*^10^5^ cells per 35 mm dish) were transfected with pCMV5-Flag PML III or pCMV5-Flag PML IV (0.8 μg/p35 mm) in the presence of NS5-GFP or GFP (0.7 μg/p35 mm). After 24 h, cells were incubated with 100 μM of cycloheximide (Sigma) for the indicated times. Proteins levels were determined by Western blot analysis. Briefly, cells were lysed at 4°C in cell lysis buffer TNN [100 mM TrisHCl (pH 8), 250 mM NaCl, 0.5% Nonidet (NP)40, and 0.1 mM DTT], with complete protease inhibitor cocktail (Roche, Basel, Switzerland). Protein extracts were quantified using the Pierce BCA Protein Assay Kit (Thermo Scientific), where 20 μg/lane was resolved by 12% SDS-PAGE, and probed with antibodies anti-FLAG M2 monoclonal antibody (Sigma-Aldrich) or anti-GFP following Western transfer. Images are representative of three independent experiments.

### Statistical Analyses

Statistical analyses were performed using GraphPad Prism software. Two sample groups were compared using student's *t* test. Groups larger than two were compared using a one-way analysis of variance (ANOVA) followed by Tukey's *post-hoc* test. Each figure legend indicates the number of samples analyzed and statistical test used.

### Safety

All work with infectious agents was performed in biosafety level 2 facilities and approved by the Office of Environmental Health and Safety at the School of Sciences, University of Buenos Aires.

## Data Availability

The raw data supporting the conclusions of this manuscript will be made available by the authors, without undue reservation, to any qualified researcher.

## Author Contributions

FG, PH, and CG contributed conception and design of the study. FG and ML performed the experiments and analyzed data. FG, ML, MM, DJ, PH, and CG wrote sections of the manuscript. All authors contributed to manuscript revision, read, and approved the submitted version.

### Conflict of Interest Statement

The authors declare that the research was conducted in the absence of any commercial or financial relationships that could be construed as a potential conflict of interest.
